# Effect of Furfurylation on Hierarchical Porous Structure of Poplar Wood

**DOI:** 10.3390/polym13010032

**Published:** 2020-12-23

**Authors:** Xiaoshuang Shen, Pan Jiang, Dengkang Guo, Gaiyun Li, Fuxiang Chu, Sheng Yang

**Affiliations:** Research Institute of Wood Industry, Chinese Academy of Forestry, Xiangshan Road, Haidian District, Beijing 100091, China; 18813089700@163.com (X.S.); panjdemo@163.com (P.J.); gdk665@hotmail.com (D.G.); ligy@criwi.org.cn (G.L.)

**Keywords:** wood, furfurylation, hierarchical pore, porosity, pore distribution

## Abstract

Some wood properties (such as permeability and acoustic properties) are closely related to its hierarchical porous structure, which is responsible for its potential applications. In this study, the effect of wood impregnation with furfuryl alcohol on its hierarchical porous structure was investigated by microscopy, mercury intrusion porosimetry and nuclear magnetic resonance cryoporometry. Results indicated decreasing lumina diameters and increasing cell wall thickness of various cells after modification. These alterations became serious with enhancing weight percent gain (WPG). Some perforations and pits were also occluded. Compared with those of untreated wood, the porosity and pore volume of two furfurylated woods decreased at most of the pore diameters, which became more remarkable with raising WPG. The majority of pore sizes (diameters of 1000~100,000 nm and 10~80 nm) of macrospores and micro-mesopores of two furfurylated woods were the same as those of untreated wood. This work could offer thorough knowledge of the hierarchical porous structure of impregnatedly modified wood and pore-related properties, thereby providing guidance for subsequent wood processing and value-added applications.

## 1. Introduction

Owing to the forbidden logging of natural forests in accordance with domestic legislation [1,2] and export restrictions [3,4] on timber in many countries, fast-growing plantations will become an increasingly important resource in the wood supply in the coming decades [5,6]. Fast-growing poplar, as one of the major plantation tree species, exhibits unique intrinsic advantages over alleviating domestic shortage of wood resources [7]. However, there are some distinct disadvantages, such as low density, dimensional instability, and unsatisfactory mechanical strength [8,9]. Consequently, various wood modifications have been dedicated to improve its properties for value-added applications.

Most wood modification strategies can be divided into surface modification (e.g., hydrophobic treatment, plasma treatment) [10,11], thermal treatment (e.g., oil-heat treatment, hydrothermal treatment) [12,13], and impregnation modification (e.g., dimethyloldihydroxyethyleneurea (DMDHEU) treatment, phenol formaldehyde resin modification, furfurylation) [14,15,16]. Various impregnation methods can be classified into cell wall modification, cell lumina filling, and cell wall modification/lumina filling [17,18,19]. Although various impregnation methods improve wood properties [20,21,22,23], some methods are involved in the harmful chemicals or release of toxic gas [24,25]. These drawbacks are driving the researchers to seek less hazardous approaches for wood modification.

As one of the candidates for impregnation technologies, furfurylation is drawing growing attention from academia and industries, due to environmentally benign of furfuryl alcohol. Emission analyses showed low release of non-reacted furfuryl alcohol in the final products and no release of volatile organic compounds (poly-aromatic hydrocarbons) above normal level for wood combustion and ecotox tests suggested low eco-toxicity of leaching water to aquatic organisms [26]. Extensive studies have been reported on physical properties, mechanical properties, and modification mechanisms, etc. of wood by furfurylation [27,28,29,30]. However, little is known about the hierarchical porous structure for furfurylated wood. The hierarchical pores are more or less affected by the resin because of wide distributions of furfuryl alcohol resin in various wood tissues [31].

The aim of this study elucidated hierarchical pores of furfurylated wood. Hierarchical porous structures, especially pore structure of cell walls, in furfurylated wood could be comprehensively understood. A better understanding of pore-related properties could be gained, thereby providing meaningful guidance with processing and value-added applications, such as drying, coating, floors and musical instruments.

According to the International Union of Pure and Applied Chemistry (IUPAC) classification, wood pores are divided into three categories: macropores (pore size > 50 nm), mesopores (2 nm < pore size < 50 nm) and micropores (pore size < 2 nm). Various methods have been used to characterize wood pores [32,33,34,35]. Mercury intrusion porosimetry (MIP) is the most commonly used to analyze macropore structure, thanks to operational ease and good stability [36]. Nuclear magnetic resonance (NMR) cryoporometry, a non-destructive technique, accurately measures pore radii from a few nm up to 100 nm [37].

To investigate the effect of furfurylation on hierarchical porous structure, furfurylated wood with different weight gain percent was chosen. Pore morphology was visualized with digital microscope. Porosity and pore distribution were determined by a combination with MIP and NMR cryoporometry.

## 2. Materials and Methods

### 2.1. Raw Materials and Chemicals

Wood blocks with 20 mm radial direction (R) × 20 mm tangential direction (T) × 300 mm longitudinal direction (L) were cut from mature sapwood of 10-year-old poplar tree (*Populus euramevicana cv. I-214*) provided by Sun jiazhuang forest farm, Yi County, Hebei province, China. The wood blocks were oven-dried before furfurylation. Wood specimens associated with each of the following tests were taken from the same piece of wood block. Furfuryl alcohol (FA) (purity ≥ 97%), maleic anhydride (purity ≥ 99.5%) and sodium borate (purity ≥ 99.5%) were bought from Sinopharm Chemical Reagent Co., Ltd. (Shanghai, China) and used as obtained without further purification.

### 2.2. Preparation of Furfurylated Wood

Impregnation solutions of 10 wt% FA and 30 wt% FA were prepared with FA, 6.5 wt% maleic anhydride, 4.0 wt% sodium borate and deionized water. Wood specimens were impregnated with as-prepared solutions through vacuum-pressure process (0.1 MPa vacuum for 30 min and 0.8 MPa pressure for 3 h). Then, impregnated specimens were wrapped in aluminum foil and kept at room temperature for 24 h. In order to polymerize FA monomer in wood, specimens were heated at 103 °C for 3 h. Subsequently, specimens were oven-dried until a dry state was reached.

### 2.3. Digital Microscope

Digital microscope (VHX-6000, Keyence, Japan) was used to observe hierarchical porous structure. Wood specimens with 5 mm (R) × 5 mm (T) × 5 mm (L) were soaked in distilled water until water saturation was achieved. Radial sections of 25 μm thick were prepared by a microtome (Leica RM2245, Wetzlar, Germany). Images were collected in the transmission mode with 500× magnification.

### 2.4. Mercury Intrusion Porosimetry (MIP)

In order to quantitatively analyze porosity and pore distribution in macropores, MIP (AutoPore IV 9520, Shimadzu, Japan) was conducted. Wood strips of 1 mm (R) × 1 (T) mm × 5 mm (L) were prepared using a blade. And then, they were dried at 103 °C until a dry state. Wood specimens of about 0.6 g were tested. The pressure was in the range of 0.5~60,000 psia. Calculation methods were carried out according to the previous literature [38].

### 2.5. Nuclear Magnetic Resonance (NMR) Cryoporometry

To quantitatively analyze porosity and pore distribution in meso- and micropores, NMR cryoporometry (Niumag MicroMR-10, Shanghai, China) was selected. Wood specimens were cut into 6 mm cylinder in diameter using a blade. In order to get water-saturated specimens, they were soaked in distilled water through vacuum-pressure process (0.1 MPa vacuum for 60 min and 1.0 MPa pressure for 24 h). Then, cylindrical specimens were wiped with tissue paper to remove water on the surface without sample drying and were weighed in the balance. Afterwards, they were put into 10 mm OD NMR tubes with Teflon caps.

In order to keep a stable magnetic field, 32 °C of the magnetic unit was set, with the precision of ± 0.01 °C. In the CPMG pulse sequence, relaxation delay was 3 s; the number of echoes was 10,000; echo time was 200 s and number of accumulated scans was 16. Different freezing temperatures were used from −45 to −5 (with incremental steps; i = 5 °C), from −5 to −2 (i = 1 °C), from −2 to −1.5 (i = 0.2 °C), from −2 to 0 (i = 0.2 °C), 1, 3, 5, and 20 °C. Calculation methods referred to the previous literature [39].

## 3. Results and Discussion

### 3.1. Characterization of Pore Morphology by Digital Microscope

**Figure 1:**
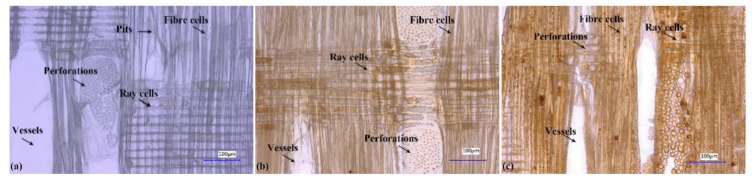
Digital microscope images of (**a**) control wood (**b**) furfurylated wood with 23% weight gain percent (**c**) furfurylated wood with 69% weight gain percent.

Wood treated with 10% and 30% furfuryl alcohol (FA) concentrations was investigated here because according to previous literature [40], no differences were seen in the locations of FA resin in wood anatomy when furfuryl alcohol content was more than 30%. Weight gain percent (WPG) of modified wood with 10% and 30% FA concentrations was 23% and 69%, respectively. Pore morphology of furfurylated wood with different WPG was shown in Figure 1. The colorless slice of poplar wood before treatment was made of tube-like vessels, thick-walled fibres and thin-walled ray cells (Figure 1a). Single vessels were linked by perforation plates, which transported fluid in vessels. One fiber cell was intercommunicated with the other fiber cell through pits. The slices of furfurylated wood with 23% and 69% WPG were reddish brown, which was attributed to distributions of colored FA resin in various cells (Figure 1b,c). The color of slices was aggravated with increasing WPG. As a result of the locations of FA resin characterized by reddish brown in tissues, it could be found that FA resin was distributed in fibres, vessels, ray cells and perforations. More importantly, a part of fiber lumina and perforations were blocked by FA resin. Pits of two furfurylated woods were not observed, which was likely attributed to pit occlusions. Cell walls of fibres became thicker and the color of the cell walls got deeper with increasing WPG, reflecting an enhancement of FA resin within cell walls with raising WPG. Thygesen et al., [31] showed that FA resin was distributed in cell walls, middle lamella, cell corners and lumina observed by confocal laser scanning microscopy. Briefly, furfurylation induced changes in pore structures, such as narrower lumina, pit occlusions and perforation occlusions. It was predictable that FA resin deposited into hierarchical pores made a contribution to the reduction of porosity.

### 3.2. Macropore Analysis by Mercury Intrusion Porosimetry (MIP)

MIP results were presented in Table 1. Both bulk density and apparent density increased after furfurylation, except for apparent density at 23% WPG. The enhancement of bulk density was 44% at 69% WPG, reflecting a reduction in wood porosity after furfurylation. However, there was a big difference in bulk density and apparent density at the same WPG. This result was probably caused by open pores [41], including lumens, pits and perforations, but excluding occluded pits and occluded perforations. Porosity was not accurate because it was recorded by MIP with pore diameters under 100 nm. Cumulative volume was used to characterize porosity with the diameters in the range of 100 nm~100,000 nm. Cumulative volume decreased as a function of WPG. The relationship could be interpreted as the increasing amount of FA resin located in macropores with increasing WPG, which was confirmed by the observed microscope results. When compared with that for untreated wood, the decrease of cumulative volume was 60% for furfurylated wood at 69% WPG.

**Figure 2:**
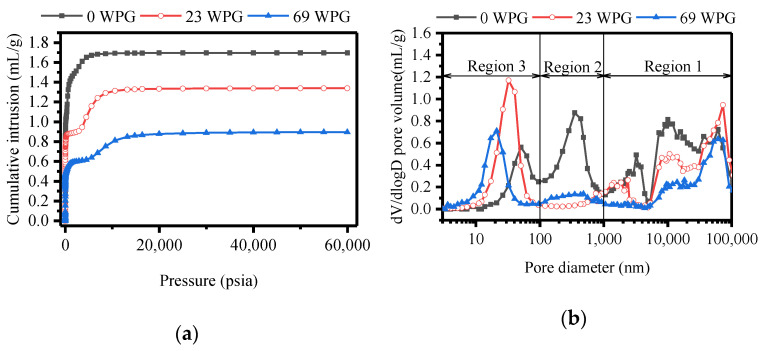
(**a**) Cumulative intrusion versus pressure (**b**) dV/dlogD pore volume versus pore size diameter in modified wood with different weight gain percent (WPG) (0%, 23% and 69%).

From Figure 2a, it could be seen that negative correlation between cumulative intrusion and WPG at the same pressure was presented. This suggested that increasing levels of furfurylation decreased porosity, which was in line with the relationship of cumulative volume and WPG. The curve of dV/dlogD pore volume versus pore diameter for unmodified wood had multimodal characteristic (Figure 2b). Pore diameters were mainly classified into three regions. Region 1 exhibited pore diameters from 1000 nm to 100,000 nm, which corresponded to the diameters of vessels lumina, fibres lumina and rays [36,42]. Region 2 displayed pore diameters between 100 nm and 1000 nm, which belonged to the diameters of longitudinal perforations in fibres/vessels, radial perforations in fibres/ vessels, pits and voids of the pit membrane [36,43]. Region 3 possessed pore diameters below 100 nm, which reflected cell wall micropores [38,44]. Furfurylation significantly changed macropore-size distributions. In region 1, dV/dlogD pore volume of most macropores was lower of two furfurylated woods than those of control sample. Moreover, this value decreased at high WPG. It was probably because FA resin was deposited into cell lumina of fibres, vessels and rays (Figure 1b,c). Increasing levels of furfurylation improved deposition of FA resin in cell lumina. The value of dV/dlogD pore volume in two furfurylated woods substantially reduced in region 2, compare to those of control. This result could be explained that pits and perforations, as one of the main pathway for liquid flow, were filled with FA solution during impregnation and then occupied by the resin through polymerization. The influence of furfurylation on macropore-size distributions was similar to that of modified wood with methyl methacrylate [36].

### 3.3. Meso- and Micropore Analysis by Nuclear Magnetic Resonance (NMR) Cryoporometry

**Figure 3:**
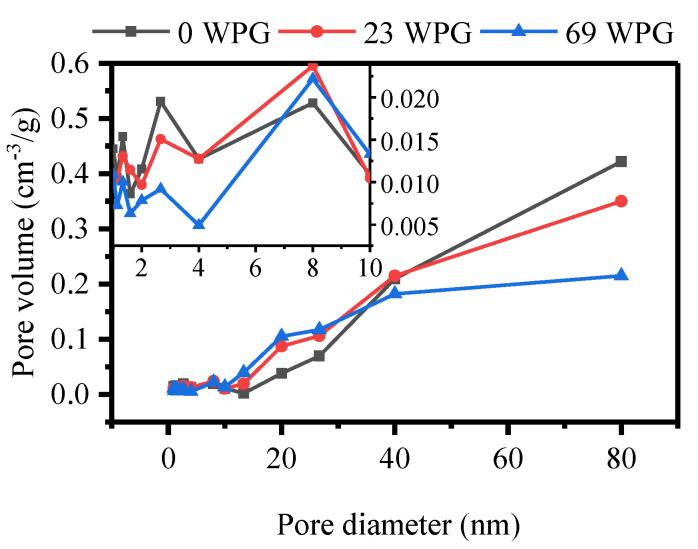
Pore volume versus pore size diameter in modified wood with different weight gain percent (WPG) (0%, 23% and 69%).

Total pore volume was shown in Table 2. Total pore volume of furfurylated wood with 23% WPG had no significant difference from that of unmodified wood. However, remarkable reduction of total pore volume at 69% WPG level was observed. This could be a result of FA resin deposited in the cell walls. Furthermore, pore size distribution was plotted (Figure 3). Pore diameters between 40 and 80 nm decreased at higher WPG. On the contrary, nearly positive correlation of pore diameters in the range of 10 and 40 nm and WPG was exhibited. It could be explained that FA resin was deposited into the larger pores, leaving less space in the range of mesopore sizes [45]. From the insert in Figure 3, it could be seen that pore volume of most pore diameters for two modified woods was smaller than that for untreated wood. This indicated that nanopore-size cell walls were infiltrated with FA resin. The result was in good agreement with the results of the observed microscope images, which showed thickener cell walls of fibres (Figure 1b,c). Because FA resin occupied nanopores within cell walls, the ability of the cell walls to accommodate water could be decrease, contributing to an improvement of dimensional stability for furfurylated wood [46,47].

Pore distribution of cell walls tested by NMR cryoporometry was obviously different from that by nitrogen sorption method, which effectively detected mesopores (2~50 nm). For nitrogen sorption, pore volume with the diameters between 2 and 10 nm was dominated and more than that with the diameters exceeding 10 nm [33,48]. Moreover, pore volume was almost zero at the diameters of 30~50 nm [33,35]. The difference of both methods could be attributable to the state of cell walls. Dried cell walls were used by nitrogen sorption while water-saturated cell walls were measured by NMR cryoporometry. When water existed, the number of voids in cell walls increased due to the bulking of cellulose [49].

### 3.4. Component of Hierarchical Pores

**Figure 4:**
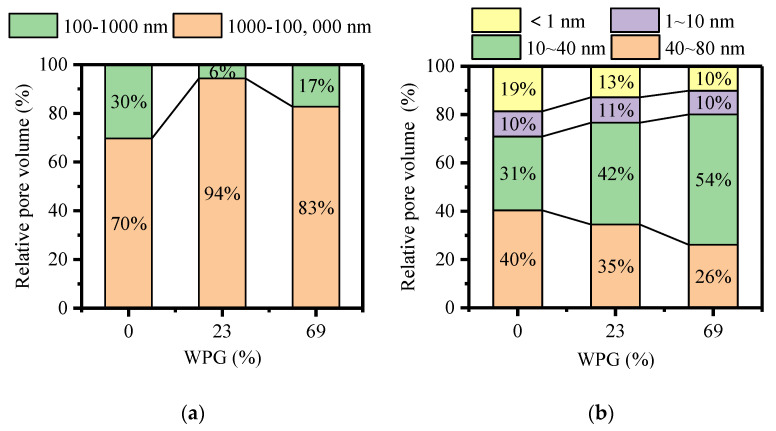
Relative pore volume of modified wood with different weight gain percent (WPG) (0%, 23% and 69%), (**a**) measured by MIP; (**b**) measured by NMR cryoporometry.

In order to further quantitatively explain the alteration of pore size of furfurylated wood, the relative pore volume was calculated by the percentage of pore volume (Figure 4). Compared with untreated wood, the relative pore volume for two furfurylated woods with pore diameters between 1000 and 100,000 nm dramatically increased and between 100 and 1000 nm notably decreased (Figure 4a). It suggested that lumina of 1000~100,000 nm made up the majority macropores of furfurylated wood, just like unmodified wood. Apparently, furfurylation had a significant influence on the relative pore volume of cell wall pores (Figure 4b). Relative pore volume shifted from pore diameters of 40~80 nm to 10~40 nm with enhancing WPG. However, cell wall pores of furfurylated wood, just as unmodified wood, mainly consisted of the sizes of 10~80 nm.

Unlike furfurylation, wax with lumina filling was only involved in a part of macropores because cell walls, resin canals, rays and axial parenchyma were difficult to wax impregnation [50,51]. Pores with the diameters less than 30 nm remarkably increased after dimethyloldihydroxyethyleneurea (DMDHEU) treatment [45]. This increase was in line with the pore diameters of 10~40 nm in this study. Although impregnation modifications made no change or an increase in some pores, the total porosity trended to a reduction, regardless of lumina filling, cell wall modification or a combination of both.

As results above, small FA molecules were impregnated into hierarchical pores of wood, including cell walls, cell lumina, pits and perforations and then formed three-dimensional polymer in these voids by in situ polymerization at high temperature. Consequently, there were some perforation and pit occlusions; narrower lumina of all type cells and thickener fibre walls were produced. Porosity and pore distribution were effected. These changes of hierarchical porous structures were likely to reduce permeability of furfurylated wood [52]. It was considered to have important influence of permeability on wood drying [53]. Perforation and pit occlusions with FA resin inevitably blocked continuous voids. Undoubtedly, the movement of free water in furfurylated wood was restricted during drying. Slow drying rates were exhibited for furfurylated wood, compared with those for unmodified wood. On one hand, the difficulty in drying of furfurylated wood was prone to generation of defects due to high stress within the wood, resulting in downgraded or rejected products. On the other hand, furfurylated wood brought about long drying time and/or high energy consuming during drying. Therefore, remarkably different from drying process of untreated wood, drying schedules of impermeable furfurylated wood should make strict measures to control quality. Besides, these results also offered a basic theory of thermal conductivity, acoustic properties, etc.

## 4. Conclusions

Hierarchical porous structures of wood were significantly influenced by furfurylation. There were alterations of hierarchical pore morphology. At the majority of pore diameters, both porosity and pore volume decreased after modification. This study gained comprehensive understanding of hierarchical porous structure for furfurylated wood and could provide meaningful guidance with subsequent processing and potential applications.

## Figures and Tables

**Table 1:** Pore structure parameters by mercury intrusion porosimetry.

Wood Types	Bulk Density/g·mL^−1^	Apparent Density/g·mL^−1^	Porosity/%	Cumulative Volume/mL·g^−1^
0 WPG ^1^	0.4004	1.2475	67.9078	1.4872
23 WPG	0.4634	1.2210	62.0478	1.3122
69 WPG	0.6097	1.3425	54.5845	0.5959

^1^ 0 WPG is unmodified wood. WPG was weight gain percent.

**Table 2:** Total pore volume by nuclear magnetic resonance cryoporometry.

Wood Types	Total Pore Volume/cm^−3^·g^−1^
0 WPG ^1^	0.8498
23 WPG	0.8845
69 WPG	0.7401

^1^ 0 WPG is unmodified wood. WPG was weight gain percent. Total pore volume was calculated by pore diameters in the range of ≤80 nm.
